# Rocky Mountain Spotted Fever Presenting as an Ischemic Stroke: A Rare Manifestation

**DOI:** 10.7759/cureus.114652

**Published:** 2026-08-17

**Authors:** Muneeb Rehman, Muhammad Daniyal, Ahmed Azam, Yasir Ahmed

**Affiliations:** 1 Infectious Diseases, Ascension St. John Medical Center, Tulsa, USA

**Keywords:** cns vasculitis, headache, ischemic stroke, rickettsia rickettsii, rocky mountain spotted fever, skin rash, thrombocytopenia

## Abstract

Rocky Mountain spotted fever (RMSF) is among the most severe tick-borne rickettsial infections encountered in the United States. Its presentation ranges from the classic triad of fever, headache, and rash to central nervous system (CNS) conditions, including meningitis, encephalitis, and seizures. Notably, RMSF presenting as an ischemic stroke due to CNS vasculitis is rare, which can prove fatal if recognition is delayed.We present the case of a woman in her 50s with a history of well-controlled diabetes mellitus type 2 and hypertension, who presented with fever, chills, body aches, and rash. She developed a headache and confusion. Routine laboratory tests showed thrombocytopenia, hyponatremia, and transaminitis. Brain magnetic resonance imaging (MRI) with and without gadolinium demonstrated scattered punctate foci of restricted diffusion throughout both cerebral hemispheres, consistent with acute lacunar infarcts involving multiple vascular territories, raising concern for vasculitis. Initial RMSF immunoglobulin G (IgG) was negative, but repeat testing showed a greater than fourfold elevation of IgG level, along with a positive plasma microbial cell-free DNA assay (Karius Inc., Redwood City, California, United States) for *Rickettsia rickettsii*. She was treated successfully with two weeks of oral doxycycline. This case highlights the importance of considering RMSF as one of the differential diagnoses when there is neurovascular involvement in a suspected tick-borne illness, especially in endemic areas. Early recognition and prompt treatment are essential to limit neurovascular injury and improve clinical outcomes.

## Introduction

Rocky Mountain spotted fever (RMSF) is caused by the obligately intracellular, gram-negative bacterium *Rickettsia rickettsii*. The organism is spread by region-specific tick vectors in the United States: the American dog tick (*Dermacentor variabilis*) in the east and the Rocky Mountain wood tick (*Dermacentor andersoni*) in the west [1]. Recently, *Rhipicephalus sanguineus* (the brown dog tick), distributed nationwide, has emerged as a new vector [2].

The clinical presentation of RMSF ranges from the classic triad of fever, rash, and headache to central nervous system (CNS) involvement [3]. *R. rickettsii* infects the endothelium of small and medium-sized vessels, producing increased vascular permeability and perivascular inflammation that leads to microvascular occlusion, the presumed mechanism of the rare neurovascular presentations of RMSF [4]. CNS ischemic stroke from vasculitis associated with RMSF is a rare and underrecognized manifestation [5]. We report a case of RMSF presenting as an ischemic multifocal stroke likely associated with CNS vasculitis.

## Case presentation

A woman in her 50s with a history of well-controlled type 2 diabetes mellitus, hypertension, hyperlipidemia, and a remote history of right-sided ischemic stroke and long-standing vertigo presented with fever, chills, body aches, and a non-pruritic rash for four days. The rash started on the abdomen and subsequently spread to the upper chest, thighs, legs, arms, hands, and back. She had recently visited California for a three-day trip, where she spent most of her time indoors with family. Before the trip started, she had removed a small tick that had been embedded for approximately two days from behind her left knee, and the above symptoms began on day 6 after tick removal.

On physical examination, her temperature was 39.9°C, heart rate was 106 beats per minute, respiratory rate was 18 breaths per minute, and blood pressure was 110/70 mmHg. A papular rash was present on the abdomen, thighs, legs, arms, upper chest, and back, but not on the palms or soles. A tick bite mark was noted in the left popliteal fossa.

Initial lab findings are shown in Table 1. Nasopharyngeal SARS-CoV-2, influenza A and B polymerase chain reaction (PCR), HIV and syphilis screens, as well as an acute viral hepatitis panel, were negative. A computed tomography (CT) scan of the abdomen with contrast demonstrated mild splenomegaly. An infectious diseases consultation was obtained, and empiric treatment with doxycycline 100 mg twice daily and ceftriaxone 2 g intravenously daily was initiated.

**Table 1:** Blood and CSF laboratory results CSF: cerebrospinal fluid; PCR: polymerase chain reaction; HSV: herpes simplex virus

Test	Result	Reference Range
White blood cell count	5.5	4 - 11.0 x 10^3^/µL
Hemoglobin	10.7	12 - 15.5 g/dL
Platelet count	36	150 - 450 x 10^3^/µL
Serum sodium	125	135 - 145 mmol/L
Aspartate aminotransferase	151	0 - 34 units/L
Alanine aminotransferase	135	0 - 55 units/L
Total bilirubin	1.7	0.3 - 1.2 mg/dL
Direct bilirubin	0.6	0.0 - 0.3 mg/dL
Serum glucose	149	70 - 100 mg/dL
CSF white blood cells	63	0 - 5 cells/μL
CSF neutrophils	70	0 - 5 %
CSF protein	63	15 - 45 mg/dL
CSF glucose	71	50 - 80 mg/dL
CSF PCR HSV Type 1 and Type 2	Negative	Negative

The patient subsequently developed confusion and headache, prompting Neurology consultation. A CT scan of the brain without contrast was unremarkable. The patient underwent a lumbar puncture. CSF studies were abnormal, as shown in Table 1. CSF bacterial culture showed no growth, and the meningitis PCR panel was negative. Serum cytomegalovirus and Epstein-Barr virus PCR were undetected. Antineutrophil cytoplasmic antibody and lupus antibody panels were negative. An initial tick-borne illness antibody panel for tularemia, RMSF, and Lyme disease was negative; however, *Ehrlichia chaffeensis* immunoglobulin G (IgG) was positive at a titer of 1:256 with a negative immunoglobulin M (IgM). *R. rickettsii* and *Ehrlichia* PCR were both negative.

**Figure 1:**
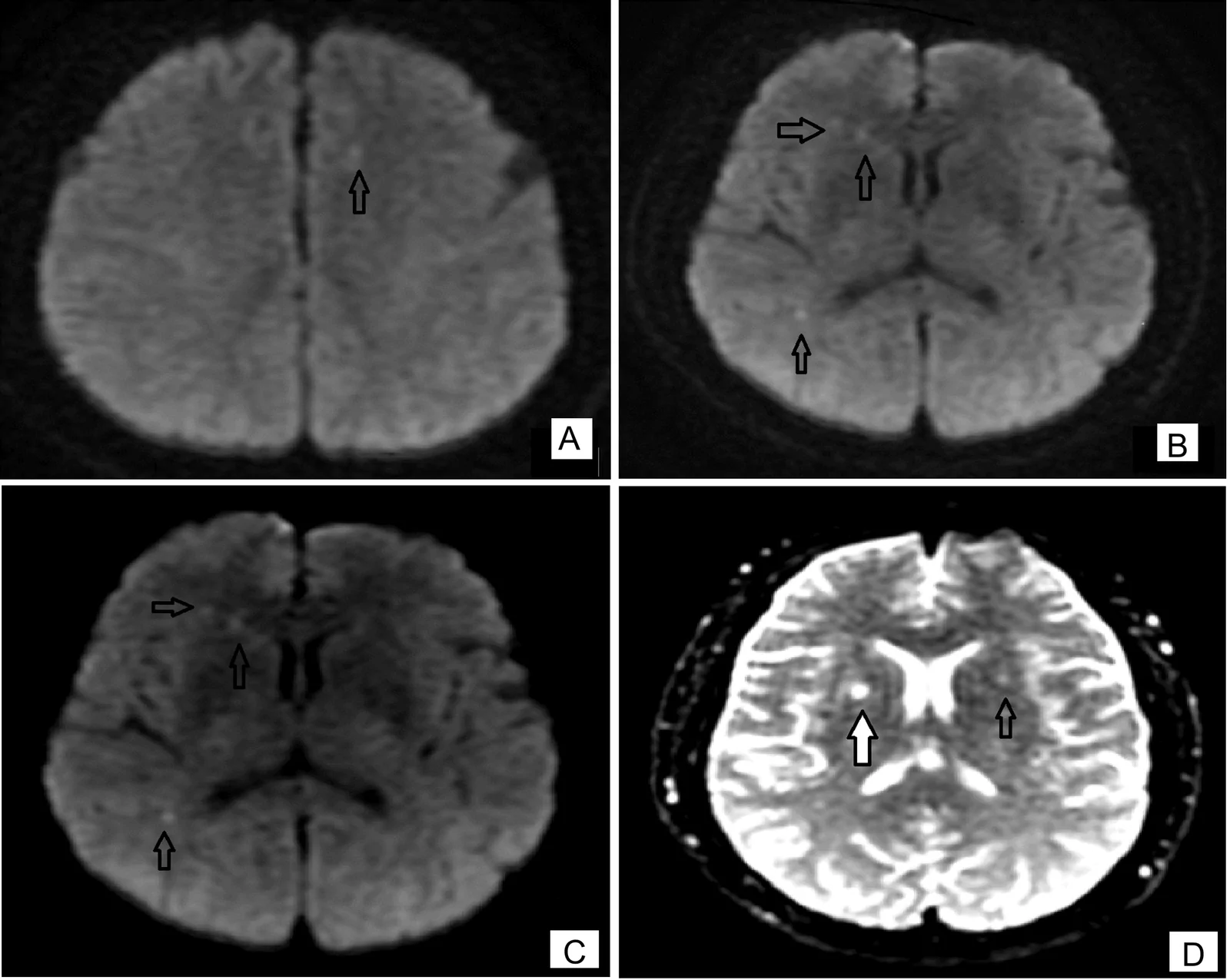
MRI of the brain with and without gadolinium contrast (axial views) (A-C) Scattered punctate areas of infarction in both cerebral hemispheres, basal ganglia, and frontal lobes are seen, consistent with acute lacunar infarction across multiple vascular territories, “starry sky pattern” (black arrow). (D) A chronic lacunar infarct in the right putamen was also noted (white solid arrow).

**Figure 2:**
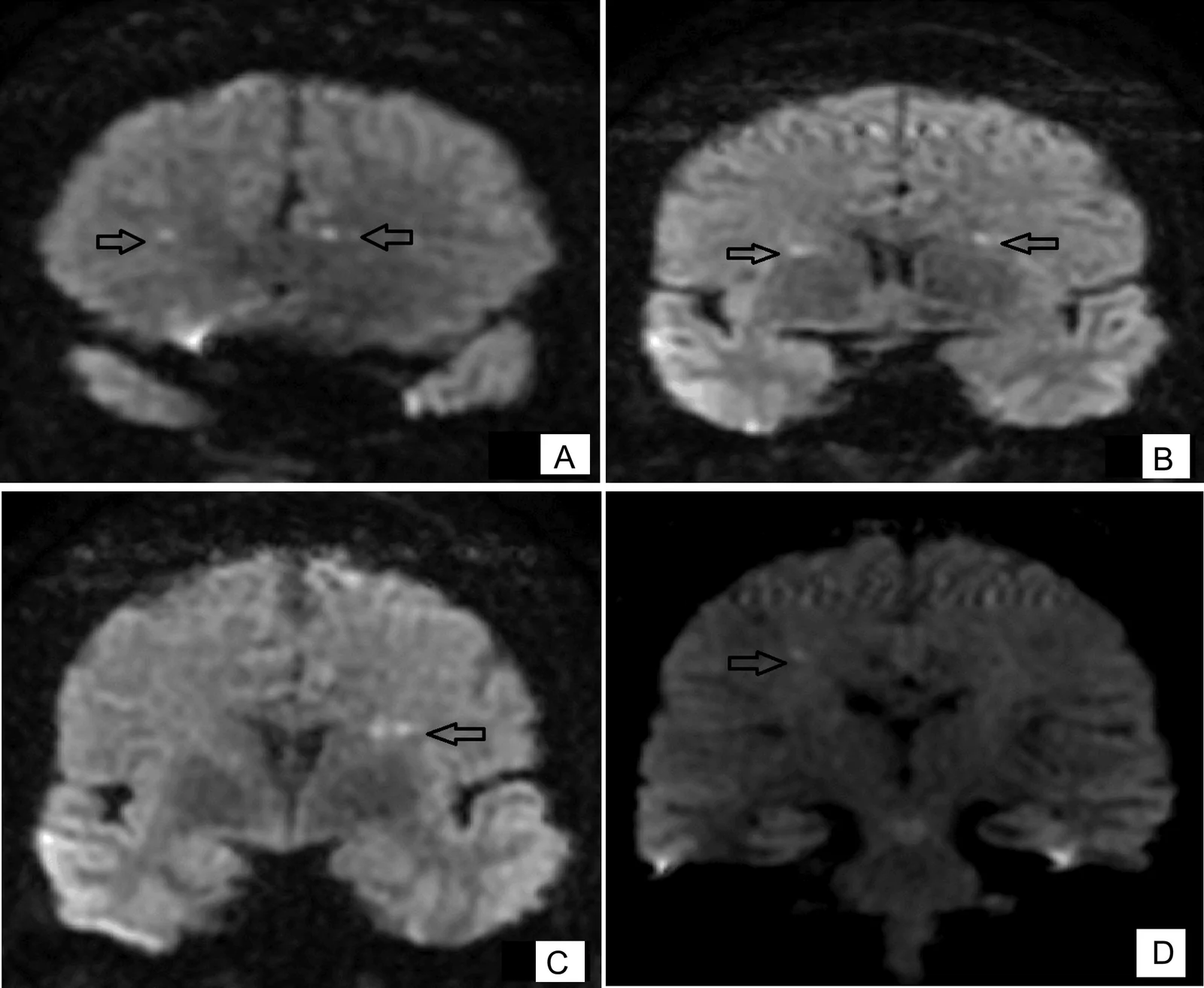
MRI Brain with and without gadolinium contrast (coronal views) Scattered punctate areas of infarctions are seen in both cerebral hemispheres, basal ganglia, and frontal lobes consistent with acute lacunar infarction across multiple vascular territories, “starry sky pattern” (black arrow).

Due to persistent encephalopathy, brain MRI with and without gadolinium contrast was performed, showing scattered punctate areas of restricted diffusion in both cerebral hemispheres, predominantly in the frontal lobes, consistent with acute lacunar infarction across multiple vascular territories, “starry sky pattern”, and raising the possibility of vasculitis (Figures 1A-1C, 2). A chronic lacunar infarct in the right putamen was also noted (Figure 1D). A CT angiography of the head and neck was negative for vasculopathy. A 12-lead electrocardiogram showed normal sinus rhythm. Transthoracic and transesophageal echocardiography showed no thrombus or vegetation; an incidental patent foramen ovale was noted. Bilateral upper- and lower-extremity venous duplex ultrasounds were negative for deep venous thrombosis. Multiple blood cultures were negative. Later, ceftriaxone was discontinued, and doxycycline was continued.

The tick-borne antibody panel was repeated on day 14 after the tick bite, at which point RMSF IgM was positive at 1:20 and IgG was positive at 1:512. At the same time, the *Ehrlichia chaffeensis *IgG titer had declined to 1:64. An isolated serum *R. rickettsii* PCR test was undetected; however, a plasma microbial cell-free DNA assay (Karius Inc., Redwood City, California, United States) confirmed the presence of *R. rickettsii* at 860 molecules per 100 nanoliters. The patient developed a post-lumbar puncture CSF leak with headache, which was treated with a blood patch. She gradually improved clinically and was discharged home on a two-week course of doxycycline. A repeat tick-borne antibody panel on day 27 after the tick bite showed an RMSF IgM titer of 1:20 and an IgG titer of 1:4096. Follow-up brain MRI at five weeks demonstrated evolving cerebral infarctions, and the patient clinically remained symptom-free.

## Discussion

RMSF is the most serious tick-borne rickettsial disease in the United States. While historically concentrated in the southeastern and south-central states, recent surveillance shows an escalated disease burden across the Southwestern United States, mirroring the high burden in Northern Mexico [6]. Drexler et al. reported that, between 2008 and 2012, 63% of RMSF cases in the United States were found in only five states: Arkansas, Missouri, North Carolina, Oklahoma, and Tennessee [7]. According to the CDC's annual cases directory in 2017, approximately 6,248 cases were reported. However, the annual number of cases has decreased since then [8].

Diagnosing RMSF in the early stages is always challenging due to its initial nonspecific clinical presentation. Illness typically begins 3-12 days after exposure, often with abrupt fever and headache alongside malaise, myalgia, nausea, and abdominal pain. A maculopapular or petechial rash may follow, characteristically appearing on the distal extremities before moving centrally [3]. However, relying on this triad of fever, headache, and rash, which isn't always present in all patients, can lead to a delayed diagnosis [9]. When the cerebral vasculature is affected, this may result in microvascular occlusion and ischemic infarctions [10]. This endothelial damage explains the characteristic cutaneous rash, multiorgan dysfunction, and neurological complications observed when the cerebral microvasculature is involved [5]. CNS involvement presents with headaches, confusion, meningitis, seizures, and encephalopathy. A frank ischemic infarction is a rare and often overlooked complication, as seen in our case [11,12].

Routine laboratory findings can also reveal hyponatremia, thrombocytopenia, anemia, transaminitis, and abnormal coagulation [9]. MRI is the modality of choice to diagnose CNS vasculitis [13]. In our patient, the distribution of infarcts favored a small-vessel rather than a single large-vessel embolic mechanism. Embolism from a proximal arterial or cardiac source characteristically produces wedge-shaped infarcts confined to one or a limited number of vascular territories. The simultaneous appearance of punctate lesions scattered across multiple territories in both hemispheres instead implies diffuse injury at the level of the cerebral microvasculature.

Common stroke etiologies were evaluated and excluded as mentioned above; CT angiogram of the head and neck was negative, and electrocardiography and transthoracic and transesophageal echocardiography showed no arrhythmias, thrombus, or vegetation. Venous duplex ultrasonography of all four limbs excluded deep vein thrombosis and also made paradoxical embolism through an incidental patent foramen ovale unlikely. Repeatedly negative blood cultures argued against infective endocarditis, and CSF meningitis PCR, CSF culture, antineutrophil cytoplasmic antibody, and lupus antibody panels were negative. After exclusion of common causes of ischemic stroke, a multifocal small-vessel pattern in the setting of confirmed rickettsial infection makes rickettsial small-vessel involvement the most likely remaining mechanism. This multifocal, punctate, diffusion-restricting pattern is consistent with the “starry sky” appearance described in prior reports of cerebral RMSF and attributed to the same small-vessel vasculitis that causes the rash [11,12,14].

This case also highlights the limitations of laboratory confirmation of RMSF. Antibodies in an indirect immunofluorescence assay (IFA) are frequently undetectable during the first week of illness [15,16]. Diagnosis is therefore best made by demonstrating a fourfold or greater rise in antibody titer between acute and convalescent specimens, as seen here when the RMSF IgG titer rose from negative to 1:512 and then to 1:4096 [16]. An initially positive *E. chaffeensis* IgG (1:256) with negative IgM and undetected ehrlichiosis PCR suggests ehrlichiosis exposure in the past or may represent cross-reactivity among tick-borne organisms. A plasma microbial cell-free DNA assay (Karius Inc.) subsequently detected *R. rickettsii*, despite negative isolated serum *R. rickettsii* serologic testing. While cell-free DNA testing can be helpful early on when standard *R. rickettsii* PCR and antibody tests come back negative, it isn't officially approved to diagnose rickettsial infections. In this case, the test supported the diagnosis; however, confirmation required a fourfold or greater increase in IgG antibody titers, the serologic gold standard for confirmation. Given that serologic testing is often negative early in the course of illness, treatment should not be withheld pending diagnostic confirmation when the clinical presentation is suggestive of RMSF, as delays in therapy are associated with severe complications and increased mortality [1,17].

Doxycycline is the first-line treatment for RMSF in patients of all ages, including during pregnancy, and prompt initiation is the most important determinant of outcome [1]. Treatment should continue for at least three days after the patient becomes afebrile, with most experts recommending a minimum duration of five to seven days; severe or complicated disease may require a longer course [1,18]. This recommendation is based on clinical experience, as no controlled trials have established an alternative treatment duration. For patients with a tetracycline allergy, doxycycline may still be administered with close monitoring or following rapid desensitization, depending on the severity of the allergy [19]. Although chloramphenicol is considered an alternative agent, it is less effective than doxycycline and is difficult to obtain in the United States [20]. Our patient was started empirically on doxycycline at admission, and she improved nicely, with normalization of her platelet count and fever. Given the severity of her clinical presentation, we elected to administer a two-week course of therapy. Although the prognosis of RMSF is excellent when treatment is started early, delayed recognition can permit irreversible neurovascular damage [11]. Reports of RMSF presenting with radiographically confirmed multifocal ischemic cerebral infarctions in adults are rare. To the best of our knowledge, few published cases have been supported by both plasma microbial cell-free DNA sequencing and subsequent serologic seroconversion. This case expands the limited literature by demonstrating this uncommon neurovascular presentation with multimodal microbiologic confirmation.

This report has important limitations as neither brain biopsy nor conventional cerebral angiography was performed, although CT angiography of the head and neck was negative for vasculopathy. Without histopathologic confirmation, the link to vasculitis stems from the patient's clinical and imaging findings, the ruling out of other causes, and response with appropriate antimicrobial therapy suggest RMSF associated with CNS vasculitis.

## Conclusions

RMSF should be considered in the differential diagnosis of unexplained multifocal ischemic stroke associated with CNS vasculitis in patients presenting with fever, rash, and a compatible history of tick exposure, particularly when evaluations for large-vessel, cardioembolic, and thromboembolic etiologies are unrevealing. Although RMSF may be associated with CNS vasculitis manifestations, establishing it as the exact cause requires correlation with the overall clinical presentation, supportive diagnostic findings, and response to appropriate antimicrobial therapy. Given the limited sensitivity of serologic testing during the early stages of infection, empiric doxycycline therapy should be initiated promptly when clinical suspicion for RMSF is high rather than deferred pending laboratory confirmation. Early recognition and prompt treatment are essential to limit neurovascular injury and improve clinical outcomes, as demonstrated in the present case.

## References

[1] Biggs HM, Behravesh CB, Bradley KK (2016). Diagnosis and management of tickborne rickettsial diseases: Rocky Mountain spotted fever and other spotted fever group rickettsioses, ehrlichioses, and anaplasmosis - United States. MMWR Recomm Rep.

[2] Demma LJ, Traeger MS, Nicholson WL (2005). Rocky Mountain spotted fever from an unexpected tick vector in Arizona. N Engl J Med.

[3] (2026). Centers for Disease Control and Prevention: Rocky Mountain spotted fever (RMSF), clinical signs and symptoms. https://www.cdc.gov/rocky-mountain-spotted-fever/hcp/signs-symptoms/index.html.

[4] Adem PV (2019). Emerging and re-emerging rickettsial infections. Semin Diagn Pathol.

[5] Bleck TP (1999). Central nervous system involvement in Rickettsial diseases. Neurol Clin.

[6] Foley J, Álvarez-Hernández G, Backus LH (2024). The emergence of Rocky Mountain spotted fever in the southwestern United States and northern Mexico requires a binational One Health approach. J Am Vet Med Assoc.

[7] Drexler NA, Dahlgren FS, Heitman KN, Massung RF, Paddock CD, Behravesh CB (2016). National surveillance of spotted fever group rickettsioses in the United States, 2008-2012. Am J Trop Med Hyg.

[8] (2026). Centers for Disease Control and Prevention: Rocky Mountain Spotted Fever (RMSF), data and statistics on spotted fever rickettsiosis. https://www.cdc.gov/rocky-mountain-spotted-fever/data-research/facts-stats/index.html.

[9] Gottlieb M, Long B, Koyfman A (2018). The evaluation and management of Rocky Mountain spotted fever in the emergency department: a review of the literature. J Emerg Med.

[10] Walker DH, Ismail N (2008). Emerging and re-emerging rickettsioses: endothelial cell infection and early disease events. Nat Rev Microbiol.

[11] Bradshaw MJ, Byrge KC, Ivey KS, Pruthi S, Bloch KC (2020). Meningoencephalitis due to spotted fever rickettsioses, including Rocky Mountain spotted fever. Clin Infect Dis.

[12] Dantas-Torres F (2007). Rocky Mountain spotted fever. Lancet Infect Dis.

[13] Bonawitz C, Castillo M, Mukherji SK (1997). Comparison of CT and MR features with clinical outcome in patients with Rocky Mountain spotted fever. AJNR Am J Neuroradiol.

[14] Mikhaiel JP, Parasram M, Park J (2025). Rocky Mountain spotted fever encephalitis and "starry sky" pattern on MRI: a case report. Neurologist.

[15] Chen LF, Sexton DJ (2008). What's new in Rocky Mountain spotted fever?. Infect Dis Clin North Am.

[16] (2026). Centers for Disease Control and Prevention: Rocky Mountain Spotted Fever (RMSF), clinical and laboratory diagnosis for Rocky Mountain spotted fever. https://www.cdc.gov/rocky-mountain-spotted-fever/hcp/diagnosis-testing/index.html.

[17] Kirkland KB, Wilkinson WE, Sexton DJ (1995). Therapeutic delay and mortality in cases of Rocky Mountain spotted fever. Clin Infect Dis.

[18] Ho BM, Davis HE, Forrester JD (2021). Wilderness Medical Society Clinical practice guidelines for the prevention and management of tick-borne illness in the United States. Wilderness Environ Med.

[19] Stollings JL, Chadha SN, Paul AM, Shaver CM, Hagaman D (2014). Doxycycline desensitization for a suspected case of ehrlichiosis. J Allergy Clin Immunol Pract.

[20] Holman RC, Paddock CD, Curns AT, Krebs JW, McQuiston JH, Childs JE (2001). Analysis of risk factors for fatal Rocky Mountain spotted fever: evidence for superiority of tetracyclines for therapy. J Infect Dis.

